# Geographic inequalities in need and provision of social prescribing link workers a retrospective study in primary care

**DOI:** 10.3399/BJGP.2023.0602

**Published:** 2024-07-09

**Authors:** Anna Wilding, Matthew Sutton, Efundem Agboraw, Luke Munford, Paul Wilson

**Affiliations:** Health Organisation, Policy and Economics, University of Manchester, Manchester, UK.; Health Organisation, Policy and Economics, University of Manchester, Manchester, UK; honorary professorial fellow, Centre for Health Economics, Monash University, Melbourne, Australia.; Health Organisation, Policy and Economics, University of Manchester, Manchester, UK.; Health Organisation, Policy and Economics, University of Manchester, Manchester, UK.; Health Organisation, Policy and Economics, University of Manchester, Manchester, UK.

**Keywords:** health services research, inequalities, primary health care, social prescribing, workforce, retrospective study

## Abstract

**Background:**

Long-term health conditions are major challenges for care systems. Social prescribing link workers have been introduced via primary care networks (PCNs) across England since 2019 to address the wider determinants of health by connecting individuals to activities, groups, or services within their local community.

**Aim:**

To assess whether the rollout of social prescribing link workers was in areas with the highest need.

**Design and setting:**

A retrospective study of social prescribing link workers in England from 2019 to 2023.

**Method:**

Workforce, population, survey, and area-level data at the PCN-level from April 2020 to October 2023 were combined. Population need before the rollout of link workers was measured using reported lack of support from local services in the 2019 General Practice Patient Survey. To assess if rollout reflected need, linear regression was used to relate provision of link workers (measured by full-time equivalent [FTE] per 10 000 patients) in each quarter to population need for support.

**Results:**

Populations in urban, more deprived areas and with higher proportions of people from minority ethnic groups had the highest reported lack of support. Geographically these were in the North West and London. Initially, there was no association between need and provision; then from July 2022, this became negative and significant. By October 2023, a 10-percentage point higher need for support was associated with a 0.035 (95% confidence interval = −0.634 to −0.066) lower FTE per 10 000 patients.

**Conclusion:**

Rollout of link workers has not been sufficiently targeted at areas with the highest need. Future deployments should be targeted at those areas.

## Introduction

Long-term health conditions and social isolation are major challenges for healthcare systems.^1^^,^^2^ One policy solution being introduced in England is social prescribing, where patients are connected to activities, groups, or services within their local community.^3^ This is typically facilitated by social prescribing link workers in primary care, who work with patients to create personalised care and support plans for their health and wellbeing needs. Depending on their needs, link workers can refer patients to a range of services such as legal and benefits advice, outdoor and physical activities, volunteering, or social support. It is important to note this requires sufficient community infrastructure within the area to facilitate this.

NHS England rolled out their national social prescribing link worker programme in July 2019 as part of the NHS Long Term Plan,^4^ aiming to employ 1000 link workers by the end of 2020/2021 and refer 900 000 people by 2023/2024. This policy was further extended with a mandate that by September 2022, every primary care network (PCN) must provide a social prescribing service to their patients.^5^ There are also link workers employed outside of this scheme from additional funding from local authorities, voluntary and charity sectors, or different NHS funding streams.^6^

PCNs are a relatively new entity within England. They were first announced in 2019 as part of the NHS Long Term Plan.^4^ They constitute a group of general practices that work together for the needs of the population they serve.^7^ The networks have access to additional funding as well as sharing resources. These PCNs feed into larger sub-integrated care boards, which pre-2022 were clinical commissioning groups (106 in total).

There are known geographic inequalities in the prevalence of long-term conditions.^8^^,^^9^ The availability of health care is often found to be inversely related to such needs.^10^ The funding scheme for social prescribing link workers in primary care in England seeks to reflect these variations in need by adjusting for age and deprivation.^11^ Older and more deprived populations receive higher weights. However, these adjustments may not be sufficient. In addition, there may be variations in the capacity of systems to recruit link workers and the ability to attract them to work locally. PCN social prescribing link workers are employed through the Additional Roles Reimbursement Scheme (ARRS). This enables PCNs to recruit additional roles to help increase access for patients, but Hutchinson *et al*^12^ found that overall the scheme had not reduced deprivation-related inequality in staffing by 2022.

**Table:** How this fits in

Social prescribing link workers were proposed in the 2019 NHS Long Term Plan to address health inequalities. Using national administrative data, this study has found that the subsequent rollout of link workers has not been sufficiently targeted at areas of highest need. Areas of higher need require additional support for employing link workers to tackle health inequalities and better support population needs.

Previous research has highlighted that the uptake of social prescribing activities is higher among individuals in areas of higher socioeconomic status.^13^ However, there are potentially greater health and wellbeing returns in social prescribing activities among individuals of lower socioeconomic status.^14^ These varying returns were reflected in a social prescribing pilot in North East England, with greater reductions in healthcare costs for individuals from minority ethnic groups and those with less complex health needs.^15^

To date, social prescribing studies have been assessed on smaller pilot schemes, and it is well documented that the existing evidence base is suboptimal.^16^^,^^17^ To the authors’ knowledge, there has not been a national analysis of social prescribing within England, particularly since the introduction of link workers who are employed through PCNs. This study aims to assess the characteristics of PCNs who have the highest need for social prescribing and examine whether these are the areas in which link workers have been concentrated.

## Method

### Data

For link worker employment, information was obtained from the PCNs workforce data^18^ for 15 financial quarters between April 2020 and October 2023. These reflect the first available data point to the most up-to-date financial quarter. The number of link worker full-time equivalents (FTEs) per 10 000 registered patients within each PCN was calculated. Specifically, this variable refers to link workers who are employed by the PCNs through the ARRS.

To assess need, data were used from the General Practice Patient Survey^19^ (GPPS) from January to March 2019, before the start of the national programme to employ link workers for social prescribing. The proportion of people with at least one (self-reported) long-term condition who responded ‘No’ (rather than ‘Yes − definitely’, ‘Yes − to some extent’, or ‘Don’t know/can’t say’) to the following question: ‘In the last 12 months, have you had enough support from local services or organisations to help you to manage your condition (or conditions)?’ was used in the current study.

This measure is reported at the general practice level, and the weighted responses that adjust for age, sex, and practice size were used to limit both over-and under-representation of individuals to better reflect the population. To obtain the PCN-level average, each GP practice ‘no support’ value was weighted using registered populations. As the number and composition of PCNs changed during the study period, these PCN-specific weighted responses were calculated for each financial quarter in the study years.

Data for each financial quarter were obtained from NHS Digital on populations registered with general practices by single year of age and by lower-layer super output areas (LSOAs).^20^ LSOAs are small geographical areas containing 1000 to 3000 people. In the current study these were linked to the 2019 Index of Multiple Deprivation income deprivation domain,^21^ and a practice-level deprivation measure was created using shares of the population in each LSOA for each practice. To obtain a measure of rurality, the proportion of the patients who reside in rural locations was calculated, linking LSOAs to Office for National Statistics (ONS) data on 2011 rural/urban classifications.^22^ The single-year age estimates were used to calculate the proportion of patients who were aged >65 years.^20^ The ethnic group composition of the general practice was obtained from the GPPS − these are in the ONS Ethnic Group 6a classification^23^ with the following categories: White (used as the base category), Black, Asian, Mixed, and Other. Any with ‘Does not apply’ were excluded from the study. These measures were mapped to PCNs to calculate weighted averages for each financial quarter.

### Analysis

To visualise geographical variation, summary statistics of the need for support and link worker employment variables are presented in heat maps for the 106 sub-integrated care boards in England. These were calculated using weighted averages of the PCN-level data.

To describe the characteristics of PCNs that have higher proportions of individuals who reported having no support from local services or organisations, linear regression models were used. The patient characteristics considered are linked with funding adjustments for the ARRS, which are age (measured via the proportion of the population aged >65 years) and deprivation (measured by proportions living in each deprivation decile). Based on previous work,^24^ the authors hypothesised that PCNs with older and more deprived populations would have higher levels of responders reporting no support. Two additional characteristics were also included: the proportion of the population whose ethnic group was Mixed, Asian, Black, or Other; and the proportion residing in rural areas. Minority ethnic groups and rural populations are known to have worse healthcare access,^25^ and the authors expected the same might be found for support from local services.

Linear regression models were used to estimate the associations between the number of link workers and the need for support reported before the rollout of link workers. This was estimated for each quarter of the rollout between April 2020 and October 2023 and the results are presented in a coefficient plot. In a supplementary analysis, the sensitivity of the results to adjusting further for the volume of need using the proportion of patients with long-term conditions was examined.

## Results

In April 2020 (quarter [Q]1), there was an average of 0.093 FTE link workers within each PCN across England, representing 0.020 FTE per 10 000 patients (Table 1). As time progressed, this increased quarter-on-quarter, and by the end of 2020/2021 there were 831 FTE link workers within England (number of PCNs multiplied by FTE). This reflected 0.144 FTE per 10 000 patients. This figure nearly doubled by the end of 2021/2022 to 0.267 FTE per 10 000 patients, reflecting an average of 1.289 FTE within each PCN, with a standard deviation of 1.513, indicating high variability. By the end of the study period (2023/2024 Q3), this further increased to 0.449 FTE per 10 000 patients, with an average of 2.198 FTE per PCN.

**Table 1.** Summary statistics of link worker employment from financial quarters from April 2020 to October 2023 across PCNs^a^

**Year and financial quarter**	**FTE, mean (SD)**	**FTE per 10 000, mean (SD)**	**PCNs, *n***
**2020/2021**			
Q1	0.093 (0.337)	0.020 (0.071)	1186
Q2	0.355 (0.775)	0.074 (0.147)	1203
Q3	0.493 (0.872)	0.102 (0.165)	1215
Q4	0.683 (1.026)	0.144 (0.201)	1217

**2021/2022**			
Q1	0.878 (1.259)	0.182 (0.236)	1218
Q2	1.009 (1.325)	0.208 (0.251)	1231
Q3	1.142 (1.404)	0.236 (0.265)	1233
Q4	1.289 (1.513)	0.267 (0.284)	1233

**2022/2023**			
Q1	1.392 (1.587)	0.287 (0.294)	1233
Q2	1.512 (1.725)	0.311 (0.311)	1254
Q3	1.625 (1.800)	0.337 (0.336)	1257
Q4	1.861 (1.886)	0.385 (0.347)	1258

**2023/2024**			
Q1	2.089 (2.062)	0.434 (0.367)	1259
Q2	2.164 (2.073)	0.444 (0.370)	1268
Q3	2.198 (2.098)	0.449 (0.372)	1270

a *Data reflect all the PCNs in England that existed in each quarter. NHS England have confirmed the accuracy of these employment numbers only from Q4 of 2022/2023.^26^ FTE = full-time equivalent. PCN = primary care network. Q = quarter. SD = standard deviation.*

**Figure 1.**
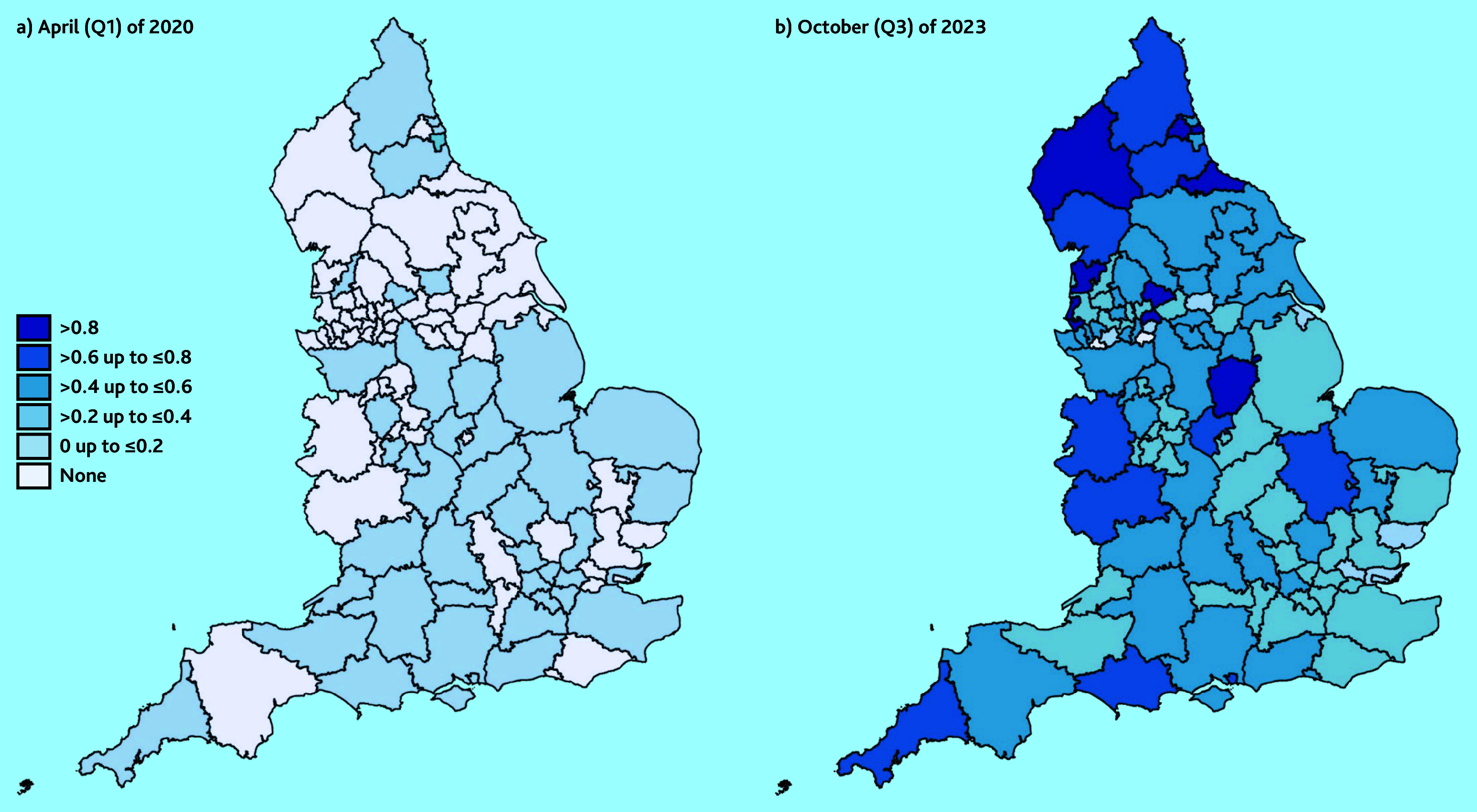
Link worker employment (FTE per 10 000 patients) by sub-integrated care board areas in England. a) April (Q1) 2020; and b) October (Q3) 2023. FTE = full-time equivalent. Q = quarter.

The standard deviations are greater than the means for all but the 2023/2024 observations (Table 1); this highlights high variability across PCNs. This is reflected in Figure 1, which shows heat maps of link worker employment across England at the start (Figure 1a) and end (Figure 1b) of the study period. There were low levels of link worker provision in April 2020 (Figure 1a), with areas in the South and North East of England having >0 FTE per 10 000 patients. The remaining areas had no link workers.

By the end of the study period, in October 2023 (Figure 1b), there had been rising employment of link workers across England. Areas in the North of England had clusters of high employment of link workers, with ≥0.8 FTE per 10 000 patients. Parts of the South East and North West of England had the lowest levels of link worker employment with a number of sub-integrated care boards, with ≤0.2 FTE per 10 000 patients. This suggests that uptake has been low since April 2020.

**Figure 2.**
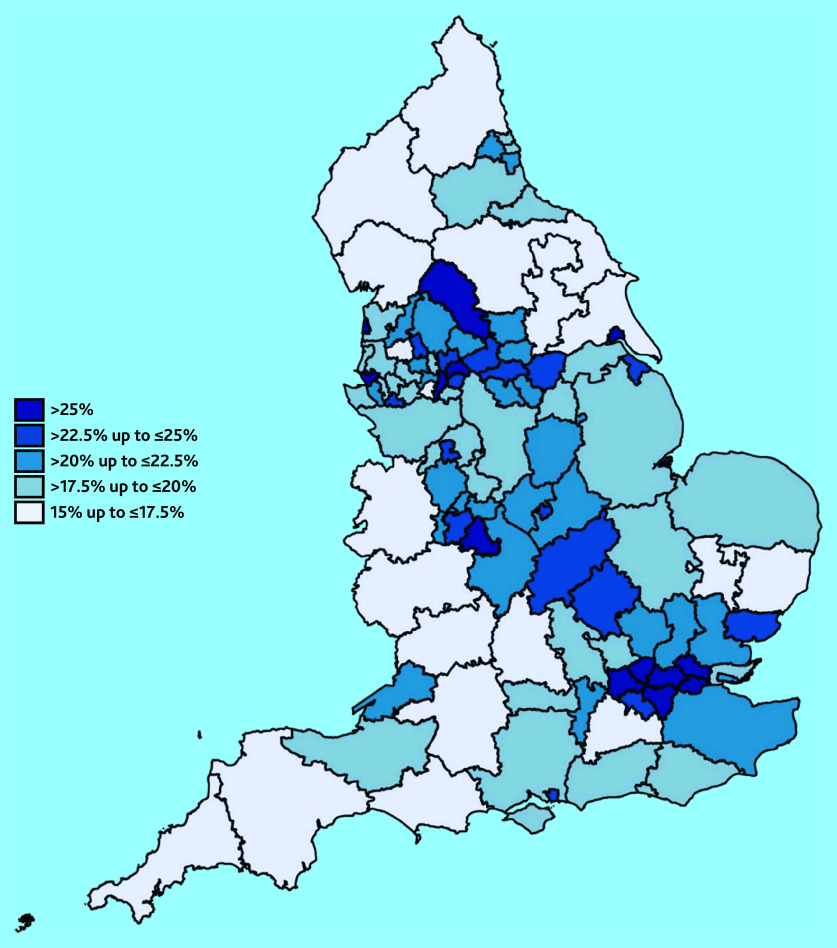
Proportions of people with at least one (self-reported) long-term condition who responded ‘No’ when asked ‘In the last 12 months, have you had enough support from local services or organisations to help you to manage your condition (or conditions)?’ by sub-integrated care board areas in England.

The values of the need for support variable across sub-integrated care boards are presented in Figure 2. There is a diagonal strip of sub-integrated care boards from the North West to the London region, where more responders reported not having support for their condition from local services or organisations. Areas in the North East and South West had the lowest proportion of individuals who reported not having enough support from local services.

Table 2 shows the characteristics of areas associated with higher levels of insufficient support from local services or organisations for their long-term conditions. PCNs with a higher proportion of patients aged >65 years had lower proportions of patients with long-term conditions feeling unsupported. PCNs with higher proportions of patients in Black (+0.258, 95% confidence interval [CI] = 0.175 to 0.342) and Asian (+0.078, 95% CI = 0.050 to 0.107) ethnic groups reported higher rates of no support from local services. PCNs serving rural areas had lower rates of not enough support reported (−0.026, 95% CI = −0.040 to −0.012) than urban areas. There is a deprivation gradient, with the least deprived areas having a lower need for social prescribing. However, this effect is not statistically different from the most deprived areas for deciles of ≤5.

**Table 2.** Regression analysis of PCN characteristics on no support

**Characteristic**	**Mean**	**No support, robust SE (95% CI)**
**Proportion of population aged >65 years**	0.182	–0.105^a^ (−0.203 to −0.007)

**Proportion of population by ethnic group**		
White (base category)	—	—
Mixed	0.020	–0.177 (−0.433 to 0.079)
Asian	0.105	0.078^b^ (0.050 to 0.107)
Black	0.043	0.258^b^ (0.175 to 0.342)
Other	0.023	0.023 (−0.151 to 0.197)

**Rurality**	0.179	–0.026^b^ (−0.040 to −0.012)

**IMD decile, income domain**		
1 — most deprived (base category)	—	—
2	0.102	–0.002 (−0.014 to 0.011)
3	0.101	0.001 (−0.013 to 0.015)
4	0.099	–0.012 (−0.026 to 0.001)
5	0.101	–0.008 (−0.022 to 0.005)
6	0.102	–0.017^a^ (−0.031 to −0.003)
7	0.100	–0.031^b^ (−0.045 to −0.017)
8	0.101	–0.034^b^ (−0.048 to −0.021)
9	0.098	–0.033^b^ (−0.047 to −0.019)
10 — least deprived	0.098	–0.054^b^ (−0.070 to −0.038)

**Constant**	—	0.238^b^ (0.215 to 0.261)

a P*<0.05.*

b P*<0.001.*

*Observations,* N *= 1270. IMD = Index of Multiple Deprivation. PCN = primary care network. SE = standard error.*

**Figure 3.**
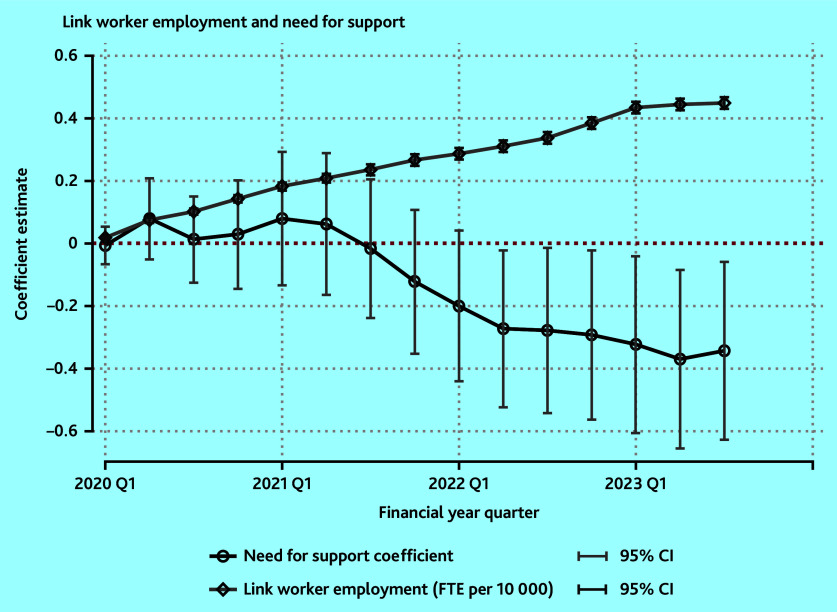
Coefficient plot of rollout of FTE link workers per 10 000 patients from Q1 of 2020 to Q3 of 2023. 95% CIs are calculated on robust standard errors. Link worker employment refers to the constant in the model. NHS England have confirmed the accuracy of these employment numbers only from Q4 of 2022/2023.^26^ FTE = full-time equivalent. Q = quarter.

Figure 3 depicts the coefficient plot from the quarterly regression models of link worker employment on the need for support. The table of results for this model is in Supplementary Table S1. There is an upward trend, as expected, in the number of link workers across the study period.

The opposite can be said for the coefficient on the need for support; this is interpreted as PCNs with higher levels of no support having decreasing levels of link worker employment during the study period. Initially, this trend is flat, from Q1 of 2020 to Q2 of 2021. This is at the start of the rollout of link workers. This trend begins to slope downwards from this point. However, it does not become statistically significant until a year later, in Q2 of 2022 (Figure 3).

From this point, the coefficients are significant, indicating lower link worker employment in areas of higher need. The coefficients from Q2 of 2022 to Q3 of 2023 begin to flatten and show this sustained difference in employment in link workers (Figure 3). As of October (Q3) 2023, for a PCN with a 10-percentage point higher need for support than another, this is reflected in a 0.035 (95% CI = −0.634 to −0.066) lower employment of FTE link worker per 10 000 patients (mean value 0.449) (see Supplementary Table S1).

The sensitivity analysis is contained in the Supplementary Table S2; the findings are robust to changing the outcome to adjust for volume so that it is the FTE per 10 000 patients with long-term conditions.

## Discussion

### Summary

This study found that areas that require additional support from services such as social prescribing had lower levels of link worker employment across England. Those PCNs that require support are from more deprived and urban areas, with a higher population of Black and Asian ethnic minorities. Despite the funding for social prescribing being adjusted for deprivation^11^ and the 2022 mandate,^5^ the current study’s findings indicate that this may not be the case and supports past research that PCNs have not changed the inequalities across England.^12^ Pre-existing health inequalities may be exacerbated by an inequitable distribution in the primary care workforce as a consequence.^27^^,^^28^

### Strengths and limitations

Administrative data were used to report the employment of social prescribing link workers within primary care in England. This allowed for a national analysis of the supply of link workers on PCN-level characteristics. This has built on past research, which has been smaller pilot studies within England.^16^

Not all PCNs were reporting their staff in the early quarters of the study. NHS England stated these employment numbers are accurate from Q4 of 2022/2023.^26^ This is evident in the results, in that the coefficients did not become statistically significant until Q2 of 2022/2023.

The measures for the outcome and explanatory variable have some drawbacks. The outcome measure does not measure the quality of the link workers or the onward referrals, nor the quantity of the local services that deliver them. The explanatory variable, the need for support, is quite general, and the question does not specifically discuss social prescribing. This has implications for findings, in that increasing employment of link workers in areas of higher needs for support may not be the only mechanism to improve service-user outcomes.

In the current study, only link workers who were funded via the ARRS were looked at. This means there could be additional link workers in the area who were funded from other sources, such as via GPs or local authorities. This means the study could be over-or underestimating the true effects and that there could be additional or fewer inequalities in areas of higher need.

### Comparison with existing literature

The current findings highlight issues of inequity around social prescribing activities. This study found lower link worker employment in areas with higher need, which are generally composed of areas of low socioeconomic status. Past findings highlight that there is a lower uptake of social prescribing activities for those service users in more deprived areas,^13^ despite greater returns to both health and wellbeing.^14^ Similarly, a study by Wildman and Wildman found that a lack of local culturally appropriate services for Black and Asian minorities limited their capacity to benefit from service provision.^15^ The study found that older populations had lower levels of no support from organisations/services before the rollout of link workers. This is contradictory to what the current study hypothesised, as the authors expected them to have higher needs as the PCN and ARRS funding is adjusted to account for this.^11^ This could be because practices are already supporting this group for their long-term conditions, or it may reflect differences in expectations. For social prescribing to deliver an equitable response to health, additional support is required for areas of higher need.

The current findings support the notion that the availability of health care is inversely related to need^10^ and that despite funding being adjusted for age and deprivation,^11^ this may not address these issues. The results are comparable with Hutchinson *et al*,^12^ who found that PCN staffing has not reduced deprivation-related inequality, which could be linked to capacity to recruit and/or ability to attract new staff to work in the local area. This is demonstrated geographically, with areas of the South East and North West of England having low uptake of link workers across the study period.

### Implications for research and practice

The findings suggest the NHS did not meet their target of employing 1000 link workers by 2020/2021^4^ (Table 1: Q4 of 2020/2021); although, it was found that each PCN was offering a social prescribing service, as per the 2022 mandate.^5^ However, there are geographical inequalities in provision, with some areas of England having very low levels of link worker employment, as shown in Figure 1b. With the results highlighting link workers being rolled out in areas of lower need, this highlights the necessity for a more targeted approach. This could be achieved through understanding the local PCN needs better and what their barriers are to employing link workers.

PCNs covering deprived areas require additional support to recruit and retain link workers. However, funding link workers is only one part of the issue. For an optimal social prescribing pathway to function, adequate voluntary and charity sector infrastructure is necessary to support local population needs. PCNs need to work closely with the local community to identify sources of support and services that match patients’ needs. These onward referral destinations also need to be funded not only to meet the demand for services but also to ensure the long-term economic sustainability of social prescribing as a whole.

## Data

Data available on request to the corresponding author.
